# Design and Synthesis of Novel 1-hydroxy-2,4,5-triaryl Imidazole Derivatives as Anti-cytokine Agents

**DOI:** 10.22037/ijpr.2019.1100909

**Published:** 2020

**Authors:** Zahra Haghighijoo, Omidreza Firuzi, Savis Meili, Najmeh Edraki, Mehdi Khoshneviszadeh, Ramin Miri

**Affiliations:** a *Medicinal and Natural Products Chemistry Research Center, Shiraz University of Medical Sciences, Shiraz, Iran. *; b *Department of Medicinal Chemistry, Faculty of Pharmacy, Shiraz University of Medical Sciences, Shiraz, Iran.*

**Keywords:** Anti-cytokine, IL-1β, Imidazole inhibitor, Inflammation, Interleukin, P38 MAPK

## Abstract

Among recent advances in the identification of anti-inflammation agents, anti-cytokines (like Interleukin-1), related to p38 MAPK families play an important role; Here in we designed new effective and low toxic anti-cytokine agents based on 1-hydroxy-2,4,5-triaryl imidazole derivatives. The reaction of oximoinoketone intermediate with ten different aromatic aldehyde and ammonium acetate in refluxing acetic acid condition give imidazole derived product, the IL-1β inhibitory assay were performed on Human PBMCs (peripheral blood mononuclear cells) using an enzyme-linked immunosorbent assay (ELISA) kit and then in computational part the binding mode of the best compound was accomplished by docking in Crystal structure of p38 MAP kinase (PDB ID: 1A9U) compared with SB202190 as standard drug. All compounds were synthesized and evaluated in biological assay showing the inhibitory activity from 28% to 82% compared to SB202190 and binding mode analysis revealed that the hydrogen-bond interactions with residues (Met109, Val30) were key point in inhibitor binding. Compound **5g** clearly proved the best inhibitory action and could be further utilized for designing newer anti-cytokine agents and p38α MAP kinase potentially inhibitory action.

## Introduction

**Figure 1:**
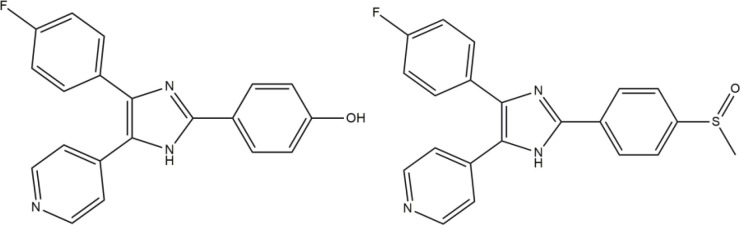
Structure of p38α inhibitor (SB-202190 and SB-203580).

One of the challenging goals in medicinal chemistry is drug discovery of safer anti-inflammation agent. Recent evidence suggests that inhibition of the p38 mitogen activated protein kinase (p38 MAPK) may be a promising approach for controlling inflammation. P38MAPK consists of four different isoforms α, β, γ, and δ. p38α is believed to be the predominant isoform involved in the inflammatory response (1, 2). The most important proinflammatory mediators, regulated by p38 include cytokines interleukin 1-beta (IL-1b) and tumor necrosis factor alpha (TNF-a) (3-5). Anakinra (Kineret) as the first anti-IL-1 agent for treatment of rheumatoid arthritis (RA) is now available on the market (6). The inhibitor that can selectively block p38 will prevent producing these destructive cytokines and ultimately alleviate the onset of inflammatory disease, such as RA, inflammatory bowel disease (IBD), psoriasis, stroke, myocardial ischemia, alzheimer disease, and respiratory disease (7, 8). The researches for finding the small molecule inhibitors are continued. Moreover, Several novel structures of imidazole analogues such as phenoxypyrimidine (9), pyridinylimidazoles (10, 11), pyrazolo (12), and pyridines (1) have shown clinical importance through exhibition of p38 inhibitor activity (13), among them some members of the pyridinyl-imidazole class have advanced into clinical trials as probable therapies for the treatment of RA. Recently, SB-202190 was revealed as a potent inhibitor of IL-1 and TNF production in human monocytes that subsequent identification of p38 as its target and also a typical p38α inhibitor like SB-203580 inhibits the enzyme with 48 nM as IC_50_ (Figure 1). The imidazole derivatives are known as competitive inhibitors at the ATP binding site of the p38α MAP kinase (14, 15).

**Figure 2.**
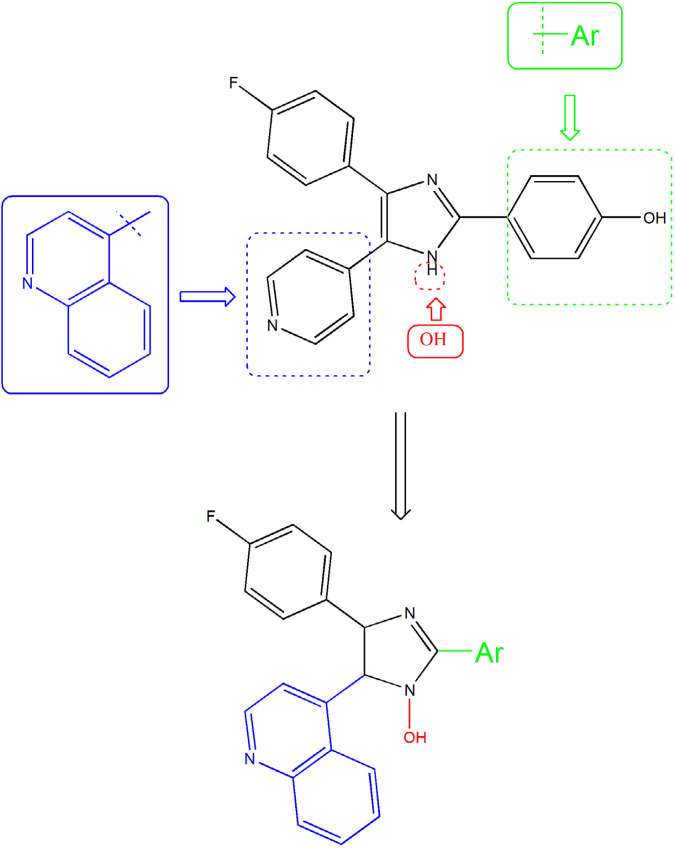
Rationale for the design of 1-hydroxy-2,4,5-triaryl imidazole (5a-5j) as anti-cytokine agents

The information from X-ray crystallography of the 4-(pyridin-4-yl)-5-phenylimidazole substructure with p38 depicted an H-bond between the nitrogen of pyridinyl and the amide NH of Met109 in the adenine binding pocket of ATP. This H bond is necessary for inhibition and also reported that is an important factor contributing to P450 inhibition (16-18). Unfortunately, that compounds that inhibited p38α, showed significant hepatotoxicity effects, thus none of them were approved to be a safe and effective drug (19-21). We aimed for the rational design of imidazole derivatives based on SB202190 scaffold with the purpose of optimizing physicochemical properties and anti-cytokine potential properties of this backbone (22). To this end, we replace H of N1 of SB202190 with OH group to facilities appropriate interaction and the introduction of the quinolone in C_5_ to enhance the accessibility and orient to the sub pocket of the active site. In addition, the role of incorporation of different aldehydes (aryl group (Ar)) at the 2-position of the imidazole were employed to investigate the importance of flexibility (Figure 2).

## Experimental

*Chemistry*

*Reagents*

All commercially available reagents and solvents were used without further purification from commercial sources. Reactions were routinely monitored by thin-layer chromatography (TLC) in silica gel (F245 Merck-Kieselgel 60 HF254, Art. 7739). IR spectra were obtained on a FT-IR Perkin-Elmer Precise system spectrophotometer (potassium bromide disks), ^1^HNMR spectra were measured on Brucker DPX-250 MHZ in δ scale. The spectra were obtained with solutions of CDCl_3_ or DMSO-d_6_ with TMS as internal standard, and the values of the chemical shifts (δ) were given in ppm, coupling constants (J) given in Hz. The mass spectra were run on Hewlett Packard (HP) 6890. 

*General chemistry procedures for 1-(4-fluorophenyl)-2-(hydroxyimino)-2-(quinolone-4-yl) ethanone (4)*

**Scheme 1:**
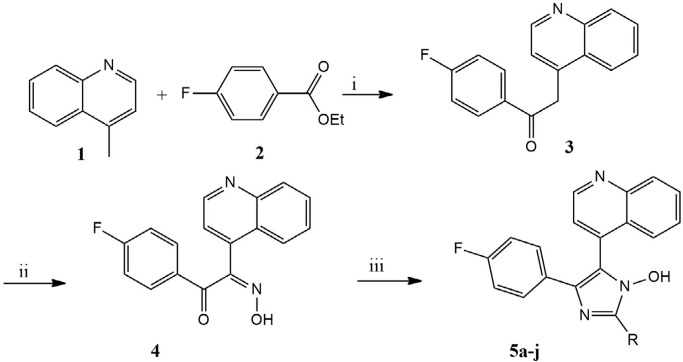
Synthetic pathway of 1-hydroxy-2,4,5-triaryl imidazole **5a-j**. Reagents and conditions (i): NaHMDS, dry THF, NaNO_2_, dry THF; (iii): ArCHO, NH_4_OAc, AcOH glacial

A mixture of 4- methylquinoline (lepidine) (**1**) (143 mg, 100 mmol) and ethyl 4- fluorobenzoate (168 mg, 100 mmol) stir at 0 °C in dry THF. In this condition 100 mL of sodium bis (tri methylsiyl) amide (NaHMDS) 2 M were added drop wise in 20 min and continued stirring about 2 h, after that reaction stirred with saturated NaNO_2_ in room temperature for 24 h. The mixture was washed with 30 mL water. The organic layer was dried over anhydrous MgSO_4_ .The solvent was then removed and the yellow pale residue was the desired compound. The Scheme 1 outlines a synthetic strategy for assembly of the substituted imidazole scaffold.

Yield: 73%. Mp: 214-216 °C. ^1^H NMR (CDCl_3_): δ ppm 13.05 (s, 1H), 9.0092 (d, *j* = 4.3, 1H), 8.19 – 8.16 (m, 2H), 8.12 (d, *j* = 8.35, 1H), 7.82–7.79 (m, 1H), 7.64–7.59 (m, 2H), 7.52 (d, *j = *4.3, 1H), 7.45–7.41 (m, 2H); MS: m/z (%) 294.1 (M^+^41), 154.1 (9.1), 123.1 (100), 95.1 (38.18), 75.1 (11), 140.1 (1.81).

*General Chemistry procedures for 5-(4-fluorophenyl)-2-(aryl)-4-(quinoline-4-yl)-1H imidazole-1-ol (* ***5a-j*** *)*

The appropriate aldehyde (1 mmol) were added to 4 (0,294 g, 1 mmol) and ammonium acetate (3.14 g) refluxed in 20 mL glacial acetic acid for 72 h, after cooling dish in ice water, neutralized with saturated NH_4_OH and the precipitate was filtered off, washed with water and was purified by column chromatography on silica diluted with EtOAc/petroleum ether (70:30) then recrystallized from suitable solvent to give the desirable pure products **5a-j**. The data of the pure products has been shown in Table 1.

*5-(4-fluorophenyl)-2-(4-methoxyphenyl)-4-(quinoline-4-yl)-1H imidazole-1-ol (* ***5a*** *)*

The titled compound was synthesized by the described procedure from 4-methoxy benzaldehyde (136 mg, 1 mmol) as colorless yellow solid. Yield: 64%. Mp: 240-243 °C. IR (KBr): νcm^-1^ 3419.25 (OH), 2926.31 (C-H aromatic), 1609.60 (C=N imidazole), 1500.05 (C=C aromatic), ^1^HNMR (CDCl3): δ ppm 11.80 (s, 1H), 8.78-8.77 (d, *j = *4.45, 1H), 8.12-8.10 (m, 2H), 7.94-7.92 (d, *j = *8.75, 1H), 7.68-7.65 (m, 1H), 7.42-7.39 (m, 2H), 7.32-7.31 (d, *j = *4.4), 7.14-7.11 (m, 1H), 6.96-6.95 (d, *j = *8.85, 2H), 6.88-6.86 (d, *j = *8.7, 2H), 3.86 (s, 3H), MS: m/z (%) 411.4 (M^+^ 26.31), 395.4 (100), 380.3 (13.04), 350.3 (8.7), 261.1 (27.17), 154.1 (7.6), 135.1 (26.1), 119.1 (14.13).

*2-(4-chlorophenyl)-5-(4-fluorophenyl)-4-(quinoline-4-yl)-1H imidazole-1-ol (* ***5b*** *)*

The orange solid product **5b** was prepared in similar described manner using 4- chlorobenzaldehyde (140 mg, 1 mmol), Yield: 71%. Mp: 221-223 °C. ^1^H NMR: δ ppm 11.78 (s, 1H), 8.38 (d, *j = *4.39, 1H), 8.06 (m, 2H), 7.80 (d, *j = *8.68, 1H), 7.55 (m, 1H), 7.34 (m, 2H), 7.27 (m, 3H), 7.14-7.04 (m, 3H), 6.72 (m, 2H).

*2-(3-fluorophenyl)-5-(4-fluorophenyl)-4-(quinoline-4-yl)-1H imidazole-1-ol (* ***5c*** *)*

Compound **5c** was prepared by the described procedure using 124 mg (1 mmol) of 3-fluorobenzaldehyde as pale yellow solid, Yield: 58%. Mp: 238-240 °C. IR(KBr): νcm^-1^ 3420.28 (OH), 2476.17 (C-H aromatic), 1612.35 (C=N imidazole), 1587.33 (C=N imidazole), 1517.97 (C=C aromatic), ^1^H NMR(DMSO): δ ppm 12.01 (s, 1H), 9.00 (d, *j = *4.52, 1H), 8.15 (m, 2H), 8.03 (d, *j = *7.86, 1H), 7.94 (m, 2H), 7.81 (m, 1H), 7.68 (m, 2H), 7.53 (m, 1H), 7.36 (m, 2H), 7.31 (m, 1H), 7.04 (m, 2H), MS: m/z (%) 399.3 (M^+^ 86.66), 382.3 (100), 261.1 (55.55), 234.2 (12.62), 107.1 (38.33).

*2-(3,4-dimethoxyphenyl)-5-(4-fluorophenyl)-4-(quinoline-4-yl)-1H imidazole-1-ol (* ***5d*** *)*

Compound 5d was obtained according to the described general procedure using 3,4 dimethoxybenzaldehyde (166 mg, 1 mmol) as orange oily, Yield: 49%. IR(KBr): νcm^-1^ 3394.15 (OH), 2926.70 (C-H aromatic), 1680.84 (C=N imidazole), 1509.66 (C=C aromatic), ^1^H NMR (CDCl_3_): δppm 12.01 (s, 1H), 8.50 (d, *j = *4.28, 1H), 8.04- 8.02 (m, 2H), 7.84-7.73 (m, 3H), 7.67-7.62 (m, 2H), 7.35 (d, 1H), 7.19-7.10 (m, 2H), 6.91-6.68 (m, 2H), 3.99 (s, 6H); MS: m/z (%) 441.4 (M^+^ 30), 425.4 (100), 408.3 (13.33), 261.1 (17.77), 165.2 (17.77).

*5-(4-fluorophenyl)-2-phenyl-4-(quinoline-4-yl)-1H imidazole-1-ol (5e)*

The titled compound 5e was synthesized according to the general procedure using benzaldehyde (106 mg, 1 mmol) as pale yellow solid, Yield: 73%. Mp: 212-214 °C. IR(KBr): νcm^-1^ 3063.56 (OH), 2568.39 (C-H aromatic), 1701.77 (C=N quinoline), 1586.68 (C=N imidazole), 1497.79 (C=C aromatic), ^1^HNMR (CDCl_3_): δ ppm 12.00 (s, 1H), 8.31-8.29 (m,1H), 8.11-8.09 (m, 2H), 7.90-7.84 (m, 1H), 7.78-7.75 (m, 1H), 7.48-7.43 (m, 2H), 7.39-7.34 (m, 2H), 7.22-7.19 (m,2H), 6.92-5.87 (m, 2H), 6.66-6.61 (m,2H); MS: *m/z *(%) 381.4 ([M^+^] 86.66), 364.3 (100), 261.1 (46.66), 234.2 (11), 140.2 (11), 107.1 (12.22), 89.2 (16.60).

*5-(4-fluorophenyl)-4-(quinoline-4-yl)-2-(thiophen-2-yl)-1H imidazole-1-ol (5f)*

The titled compound 5f was synthesized according to the general procedure using thiophen-2-carbaldehyde (112 mg, 1 mmol) as brown oily, Yield: 48%. ^1^H NMR(CDCl_3_): δ ppm 11.83 (s, 1H), 8.98 (d, *j = *4.41, 1H), 8.38-8.34 (m, 2H), 8.08-8.02 (m, 1H), 7.80 (s, 1H), 7.54-7.48 (m, 1H), 7.43 (d, *j = *4.1, 1H), 7.25-7.17 (m, 2H), 7.08 (s, 1H), 7.01-6.94 (m, 1H), 6.61-6.68 (m, 2H).

*5-(4-fluorophenyl)-2-(4-hydroxy-3-methoxyphenyl)-4-(quinoline-4-yl)-1H imidazole-1-ol (* ***5g*** *)*

The yellow solid product 5g was prepared from 4-hydroxy-3-methoxybenzaldehyde (152 mg, 1 mmol) according to the general procedure, Yield: 61%. Mp: 198-200 °C. ^1^H NMR (CDCl_3_): δ ppm 11.69 (s, 1H), 9.22 (s, 1H), 8.58 (d, *j = *4.29, 1H), 7.99-8.03 (m, 2H), 7.81 (d, *j = *8.63, 1H), 7.54-7.51 (m, 1H), 7.46-7.41 (m, 2H), 7.29 (m, 1H), 7.19-7.15 (m, 2H), 6.84 (m, 1H), 6.77 (m, 2H), 3.94 (s, 3H).

*2-(4-bromophenyl)-5-(4-fluorophenyl)-4-(quinoline-4-yl)-1H imidazole-1-ol (5h)*

The titled compound 5h was synthesized according to the general procedure from 4-bromobenzaldehyde (184 mg, 1 mmol) as orange solid, Yield: 65%. Mp: 208-210 °C. IR(KBr): νcm^-1^ 3062.99 (OH), 2563.14 (C-H aromatic), 1587.84 (C=N imidazole), 1511.49 (C=C aromatic), ^1^H NMR (CDCl_3_): δ ppm 11.98 (s, 1H), 8.20 (d, *j = *3.65, 1H), 8.06-8.04 (m, 2H), 7.75 (d, *j = *7.95, 1H), 7.53-7.51 (m, 1H), 7.44-7.43 (m, 2H), 7.427-7.40 (m, 1H), 7.30-7.28 (m, 1H), 7.22-7.21 (m, 1H), 6.83 (m, 2H), 6.63 (m, 2H).

*2-(2,4-dichlorophenyl)-5-(4-flourophenyl)-4-(quinoline-4-yl)-1H imidazole-1-ol (5i)*

The titled compound 5i was synthesized according to the general procedure using 2,4-dichlorobenzaldehyde (175 mg, 1 mmol) as orange solid, Yield: 56%. Mp: 211-213 °C. ^1^H NMR (CDCl_3_): δ ppm 11.98 (s, 1H), 8.95 (d, *j = *4.27, 1H), 8.22-8.19 (m, 2H), 8.04-7.98 (m, 1H), 7.74-7.68 (m, 1H), 7.49-7.44 (m, 2H), 7.37 (d, *j = *3.9, 1H), 7.18-7.14 (m, 2H), 6.95-6.93 (m, 2H), 6.88-6.86 (m, 1H).

*5-(4-fluorophenyl)-2-(2-hydroxy-3-methoxyphenyl)-4-(quinoline-4-yl)-1H imidazole-1-ol (5j)*

Compound 5j was obtained according to the described general procedure using 2-hydroxy-3-methoxybenzaldehyde (152 mg, 1 mmol) as orange solid, Yield: 52%. Mp: 235-237 °C. ^1^H NMR (CDCl_3_): δ ppm 12.00 (s, 1H), 8.84 (s, 1H), 8.514-8.509 (m, 1H), 8.11-8.14 (m, 2H), 7.98-8.05 (m, 1H), 7.87-7.94 (m, 1H), 7.66-7.73 (m, 2H), 7.57-7.55 (m, 1H), 7.33-7.39 (m, 2H), 7.17-7.23 (m, 2H), 6-79-6.89 (m,1H), 3.79(s, 3H).

*Determination of IL-1β inhibitory*

*Reagents and method*

RPMI 1640 media, FBS (Fetal Bovine Serum), trypsin, penicillin, and streptomycin were purchased from Biosera (Austria). SB202190 and Ficoll-hypaque were purchased from Sigma (USA). ELISA kit was from Enzo® Life Science International Inc.

Inhibition of IL-1β production in a cell-based assay: Human peripheral blood mononuclear cells (PBMC) in RPMI 1640 medium containing 2.5 mM EDTA, and Ficoll-Hypaque (sigma) were overlaid with the mixture and centrifuged at 1500 rpm for 30 min, after collecting the buffy coat and cell washing, the cells was plated in 96-well plates at a concentration of 1 × 106 cells/mL in assay medium (RPMI medium containing 10% fetal bovine serum) and incubated with test compounds for 30 min and then stimulated by the addition of LPS (1 µg/mL) for a total of 6 h at 37 °C, 5% CO_2_. Following incubation, the culture medium was harvested (100 µL) and stored at -70 °C. IL-1b concentration in the medium, quantified using an enzyme-linked immunosorbent assay (ELISA) kit following the manufacturer’s instructions (assay designs). The absorbance in each well was measured with a micro plate reader at 450 nm and corrected at 570 nm. The concentrations of cytokines released were obtained and the percentage of inhibition of cytokines production was calculated as compared to the control conditions and were displayed in Table 2 (23).

*Molecular docking*

The chemical structures of the desired ligands were created using HyperChem software version 8.0.10 and energetically minimized using MM^+^ force field. Molecular docking 4 studies of the compounds 5g and SB202190 were performed using PYRX [Wolf LK, Chemical and Engineering News. 2009 87: 31]. Crystal structure of MAP kinase was downloaded from the Protein Data Bank (PDB ID: 1A9U resolution: 2.50Å). Water molecules and cognate ligand were removed from the receptor. The active sites were defined based on the center (box: x: 2.25 y: 15.49 z: 28.18). Docking computations were performed using the Lamarkian genetic algorithm, the docking procedure was conducted according to previous work and the lowest free binding energy with the most favorable docked poses was selected for analyzing enzyme-inhibitor interactions. 

**Figure 3:**
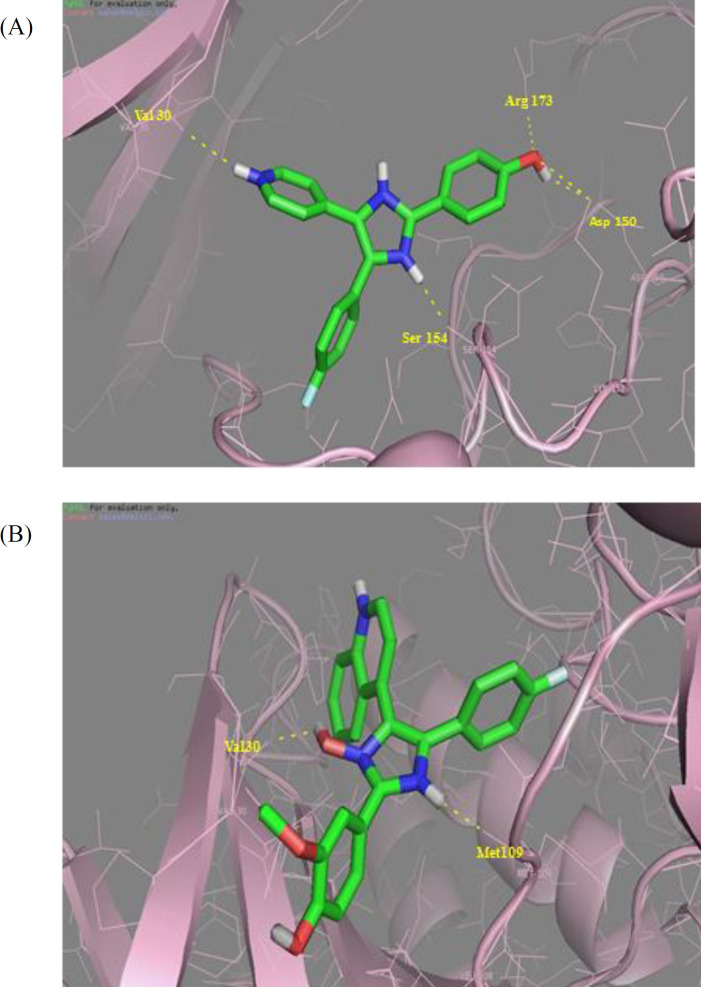
Crystal structure of p38α complexed with H bonds between Val30, Asp150, Ser154, Arg173 and (A) SB202190 and (B) **5g** that show two H bonds with Val30 and Met 109

**Table 1:** Structures and physical properties of synthesized compounds (**5a-j**)**.**

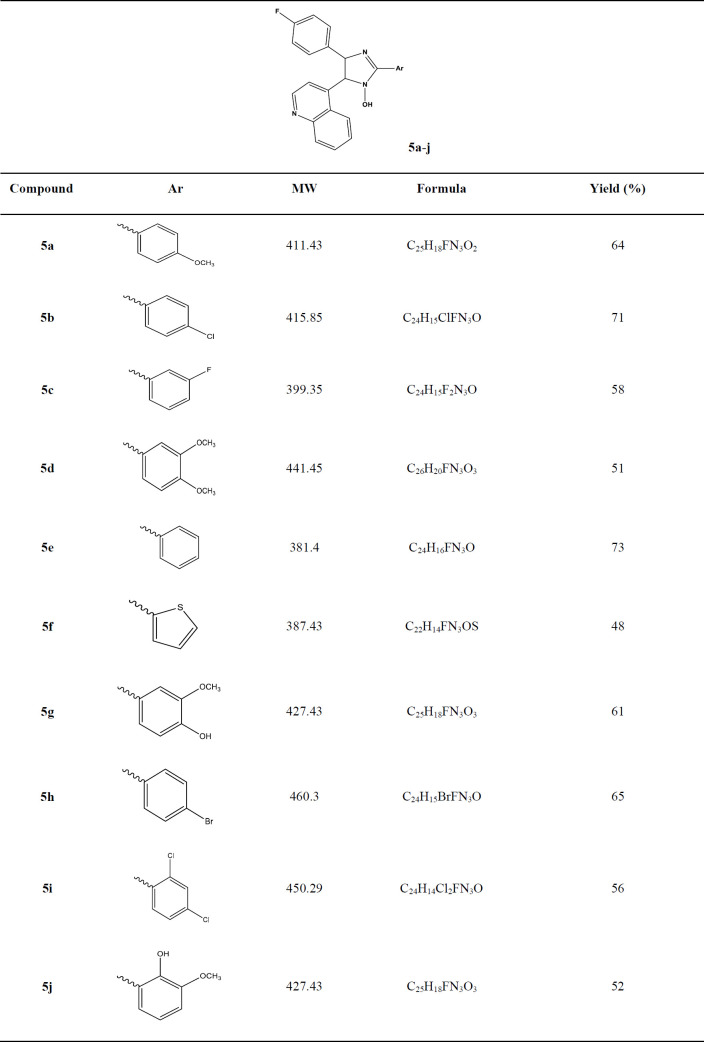

**Table 2:** The percentage of inhibition of cytokines production of compounds **5a-j**

**Compound**	**Percent ** **Inhibition at 10 μM**
**5a**	81.61% (±2.68)
**5b**	78.64% (±2.18)
**5c**	65.43% (±5.12)
**5d**	70.32% (±2.29)
**5e**	32.24% (±6.21)
**5f**	28.34% (±1.85)
**5g**	82.17% (±4.41)
**5h**	76.26% (±4.39)
**5i**	76.26% (±4.39)
**5j**	N.D
**SB202190**	94.36% (±2.26)

**Table 3:** Metabolism probabilities pathway for synthesized (**5a, b** and **g**) compounds

**Compound**	**Substrate**			**Inhibitor**				
	**CYP450 2C9**	**CYP450 2D6**	**CYP450 3A4**	**CYP450 2C9**	**CYP450 2D6**	**CYP450 3A4**	**CYP450 1A2**	**CYP450 2C19**
**SB202190**	**-**	**-**	**-**	**+**	**+**	**+**	**+**	**+**
**5a**	**-**	**-**	**+**	**-**	**-**	**-**	**+**	**-**
**5b**	**-**	**-**	**-**	**-**	**-**	**-**	**+**	**+**
**5g**	**-**	**-**	**-**	**+**	**-**	**-**	**+**	**+**

## Results and Discussion

*Chemistry*

The synthesis of the compounds concentrated on two coupling reactions, the first connected 4- methylquinoline (lepidine) (1) to a carbonyl core (2) by sodium bis (tri methylsiyl) amide (NaHMDS) gives compound (3) and then in presence of NaNO_2_ aqua solution, oximinoketone (4) formed, while the second coupled were cyclization reaction of 4 with different aromatic aldehyde and ammonium acetate in refluxing acetic acid to give imidazole derived product 5a-j.; The spectral data of synthesized compounds were in agreement with the assigned structure (24, 25). 

*Biology*

All Target compounds were evaluated for their ability to inhibit IL-1β release in lipopolysaccharide (LPS) stimulated in PBMC (peripheral blood mononuclear cells), LPS is major constituent of the outer membrane of gram-negative bacteria that induces a systemic inflammation response, and it is a well-studied immunostimulator, particularly, the expression of proinflammatory cytokines such as IL-1β representative results with their percentage for inhibition of IL-1β existing in Table 1, that have been identified exhibiting moderate to excellent inhibition in comparison to SB-202190. The substituted at various position of the pendant phenyl were chosen from potent compounds based on pharmacophore study and recent reports (26). Notably, Compound 5g bearing *para*-hydroxy-*meta*- methoxyphenyl pendant demonstrated the most inhibition percentage of cytokines production equal to 82.17% at concentrations of 10 µM. Furthermore the presence of different aryl substituted with electron-donating or electron-withdrawing group on the phenyl ring may result in improved cytokines production inhibitory activity.

In this regard, compounds such as 5a bearing *para*- methoxyphenyl counterpart (81.61% inhibition) and 5b *para*- chlorophenyl derivative (78.64% inhibition) also exhibited significant activity at the concentrations of 10 µM. The obtained results, revealed that the unsubstituted phenyl derivative 5e and thiophene 5f counterparts showed lower activity (32.2 and 28.3% inhibition) in comparison with the substituted moiety on phenyl ring.

*Molecular docking study*

To rationalize our design and our biological activity assay, docking study was carried out for the most bioactive compounds 5g and SB202190 to elucidate the molecular reasons behind the observed inhibition profile, including which amino acids in the p38α MAP kinase active site would be involved in the anticipated hydrogen bonding interactions. According to the results obtained with the rigid docking approach, SB202190 (Figure 3A) has polar interactions with Val 30, Asp150, Ser154, and Arg173. The docking poses of the novel 5-(4-fluorophenyl)-4-(quinoline-4-yl)-2-(thiophen-2-yl)-1H imidazole-1-ol compound and the p38α MAP kinase ATP binding site is depicted in (Figure 3B). Two important hydrogen bond interactions could be seen between hydrogen bond between NH and Met 109 and also OH and Val 30 of imidazole heterocyclic core. These interactions are analogous to the H-bonding interactions typically seen between the inhibitor and active site residues, while the target protein conformation studies revealed that the hydrogen-bond interactions with residues (Met109, Glu71, and Asp168) was a key point in inhibitor binding.

*In-silico metabolism prediction*

Nowadays, numerous approaches have been developed to estimate drug-likeness of bioactive compounds based on topological descriptors of molecular structure or other properties such as molecular weight, water solubility, and cLogP. In this study, the open-source program admetSAR was used to evaluate chemical metabolism properties of the most potent compounds to compare them with SB202190. The admet SAR provides a database including FDA approved and experimental investigated drugs, pesticides, environmental agents, industrial chemicals that can predict absorption, distribution, metabolism, excretion and toxicity profiles (27), here in *in-silico* prediction of CYP450 metabolism are set in Table 3. Interestingly, the designed compounds (5a, b and g) showed less interaction with different CYP450 enzyme isoforms than SB202190. This information may help us to generate a new safer compound in this category.

## Conclusion

Here, we have generated a novel molecular framework, 1-hydroxy-2,4,5-triaryl imidazole derivatives by utilizing the chemical lead based approach and concept of pharmacophore of 4-(pyridin-4-yl)-5-phenylimidazole derivatives. Compounds based on this framework have found to be as anti-cytokine and p38α MAP kinase inhibitory action with a convenient approach. The results indicated that the potent analog of this scaffold, 5g bearing para-hydroxy-meta- methoxyphenyl pendant with superior inhibitory action among all compounds could serve as a suitable candidate for further modification as a lead anti cytokine structure.
